# Identification and validation of endogenous control miRNAs in plasma samples for normalization of qPCR data for Alzheimer’s disease

**DOI:** 10.1186/s13195-020-00735-x

**Published:** 2020-12-05

**Authors:** F. Dakterzada, A. Targa, I. D. Benítez, L. Romero-ElKhayat, D. de Gonzalo-Calvo, G. Torres, A. Moncusí-Moix, R. Huerto, M. Sánchez-de-la-Torre, F. Barbé, G. Piñol-Ripoll

**Affiliations:** 1 Unitat Trastorns Cognitius, Clinical Neuroscience Research, Hospital Universitari de Santa Maria, IRBLleida, Lleida, Spain; 2 Translational Research in Respiratory Medicine, Hospital Universitari Arnau de Vilanova-Santa Maria, IRBLleida, Lleida, Spain; 3 Centro de Investigación Biomédica en Red de Enfermedades Respiratorias (CIBERES), Madrid, Spain; 4 Group of Precision Medicine in Chronic Diseases, Hospital Universitari Arnau de Vilanova-Santa Maria, IRBLleida, Lleida, Spain

**Keywords:** Alzheimer’s disease, Biomarkers, Mild cognitive impairment, miRNAs, Normalization, qPCR

## Abstract

**Background:**

MicroRNAs (miRNAs) are noncoding RNAs that are highly relevant as disease biomarkers. Several studies that explored the role of miRNAs in Alzheimer’s disease (AD) demonstrated their usefulness in clinical identification. Nevertheless, miRNAs that may act as endogenous controls (ECs) have not yet been established. The identification of ECs would contribute to the standardization of these biomarkers in AD. The objective of the study was to identify miRNAs that can be used as ECs in AD.

**Methods:**

We evaluated 145 patients divided into two different cohorts. One was a discovery cohort of 19 women diagnosed with mild to moderate AD (Mini-Mental State Examination (MMSE) score ≥ 20) and with confirmed pathologic levels of Aβ42 in CSF. The stability assessment cohort consisted of 126 individuals: 24 subjects without AD or any kind of dementia and negative for all core CSF biomarkers of AD, 25 subjects with MCI and negative for CSF biomarkers (MCI −), 22 subjects with MCI and positive for CSF biomarkers (MCI +), and 55 subjects with AD and positive for CSF biomarkers. In the discovery cohort, a profile of 384 miRNAs was determined in the plasma by TaqMan low-density array. The best EC candidates were identified by mean-centering and concordance correlation restricted normalization methods. The stability of the EC candidates was assessed using the GeNorm, BestKeeper, and NormFinder algorithms.

**Results:**

Nine miRNAs (hsa-miR-324-5p, hsa-miR-22-5p, hsa-miR-103a-2-5p, hsa-miR-362-5p, hsa-miR-425-3p, hsa-miR-423-5p, hsa-let-7i-3p, hsa-miR-532-5p, and hsa-miR-1301-3p) were identified as EC candidates in the discovery cohort. The validation results indicated that hsa-miR-103a-2-5p was the best EC, followed by hsa-miR-22-5p, hsa-miR-1301-3p, and hsa-miR-425-3p, which had similar stability values in all three algorithms.

**Conclusions:**

We identified a profile of four miRNAs as potential plasma ECs to be used for normalization of miRNA expression data in studies of subjects with cognitive impairment.

**Supplementary Information:**

The online version contains supplementary material available at 10.1186/s13195-020-00735-x.

## Introduction

MicroRNAs (miRNAs) are small (typically 22 nt in size) noncoding RNA molecules that regulate the activity of specific messenger RNA (mRNA) targets by binding to their 3′-untranslated regions (UTRs). This interaction suppresses the translation of the mRNA or induces its degradation [1]. miRNAs play important roles in a wide range of physiologic and pathologic processes [2–4]. They are present in the tissue and body fluids. Circulating miRNAs have shown high stability [5, 6], making them promising biomarker targets. Reverse transcription quantitative real-time polymerase chain reaction (RT-qPCR) is widely used for quantification of miRNA expression due to its sensitivity, specificity, speed, simplicity, and the small amount of template RNA required. However, some experimentally induced artifacts, e.g., starting sample amount, collection and storage conditions, and miRNA extraction/transcription efficiency, profoundly affect the final result of RT-qPCR, eventually affecting the interpretation of the biological response. To overcome this problem, the use of endogenous miRNAs as normalizers is the method of choice because their expression is affected by the same variables as the expression of target genes [7]. Normalization is the statistical process rating a set of candidate genes according to their expression stability in a given population and in a given experimental design. This process aims to reduce the analytical variability to obtain the most reliable and reproducible biological result [7]. Currently, mean-centering normalization is the most accurate method for normalizing high-throughput RT-qPCR data [7, 8]. However, when analyzing a small number of miRNAs, mean-centering normalization is not a valid method, and in those cases, the best strategy is the use of endogenous control (EC) genes [7–9].

Alzheimer’s disease (AD) is a progressive brain disorder caused by a gradual loss of brain cells that leads to memory loss and decline in other cognitive abilities. Extracellular plaques (deposits of amyloid-β42 protein (Aβ42)) and intracellular neurofibrillary tangles (aggregates of abnormally hyperphosphorylated tau protein) are the two neuropathological hallmarks of AD. Currently, AD is diagnosed based on cognitive assessment, PET or CSF marker (Aβ42, total-tau, and phospho-tau) measurement, and neuroimaging [10]. Cognitive tests can only diagnose the disease in the symptomatic stage when pathological events have started many years before. On the other hand, PET techniques are not cost-effective for routine use in clinical practice. Although CSF biomarkers seem to reflect the biochemical alterations occurring in the brain, a lumbar puncture to obtain CSF is an invasive procedure that limits its use for the concurrent monitoring of therapeutic trials, drug efficacy, and longitudinal studies [11]. Therefore, finding markers in the circulatory system that overcome these limitations is highly valuable. miRNAs are critically involved in different pathological processes throughout AD progression [12]. An increasing body of evidence, although inconsistent, indicates a differential expression profile of circulatory miRNAs in patients with AD compared with healthy controls [13, 14]. This inconsistency can mainly be attributed to the lack of universally accepted endogenous miRNAs suitable for data normalization. Therefore, our objective was to identify a panel of stable miRNAs (using microarray profiling) in the plasma of AD patients and then assess the stability of these selected miRNAs in the plasma of several populations along the continuum of AD disease: noncognitive impairment subjects, patients with mild cognitive impairment (MCI) with normal CSF biomarkers, MCI patients with pathological CSF biomarkers, and AD patients with pathological CSF biomarkers.

## Material and methods

### Study population

The subjects were prospectively recruited from a sample of outpatients who visited the Cognitive Disorders Unit at Hospital Universitari Santa Maria in Lleida between April 2015 and August 2017. The screening study population consisted of 19 women diagnosed with mild to moderate AD (Mini-Mental State Examination (MMSE) score ≥ 20) and with confirmed pathologic levels of Aβ42 in CSF. The stability assessment study consisted of 126 individuals as follows: 24 subjects without AD or any kind of dementia and negative for all core CSF biomarkers of AD, 25 subjects with MCI and negative for CSF biomarkers (MCI −), 22 subjects with MCI and positive for CSF biomarkers (MCI +), and 55 subjects with AD and positive for CSF biomarkers. AD and MCI were diagnosed according to the criteria of the National Institute on Aging and Alzheimer’s Disease Association (NIA-AA) [10, 15]. Patients with cognitive impairment caused by other conditions, such as stroke, brain tumor, and cortical dysplasia giving rise to epilepsy, identified by computed tomography (CT) or brain magnetic resonance imaging (MRI), or analytical alterations such as ionic alterations, hypothyroidism, deficit of cobalamin or folic acid, and positive syphilitic serology were excluded from the study. We also excluded male patients from the discovery study to eliminate the gender effect in this small population. Demographic data and general medical aspects such as hypertension, diabetes mellitus, hypercholesterolemia, depression, and MMSE were also evaluated in all subjects.

### Sample collection

Blood samples were collected by venipuncture into BD vacutainer tubes containing EDTA between 8:00 and 10:00 A.M. The samples were centrifuged at 1500×*g* for 10 min, and aliquots of the plasma were stored at − 80 °C until use. The CSF samples were collected by a lumbar puncture on the same morning when blood samples were collected and after overnight fasting. Briefly, the samples were centrifuged at low speed to pellet any cellular debris, aliquoted in polypropylene tubes, and finally, frozen at − 80 °C. CSF Aβ42, total tau (T-tau), and phospho-tau (P-tau) were measured by enzyme-linked immunosorbent assay (ELISA) (INNOTEST, Innogenetics). The cutoff values for these biomarkers were determined in an independent cohort of AD patients and controls in our laboratory. The cutoff values for Aβ42, T-tau, and P-tau were as follows: < 600 pg/ml, > 425 pg/ml, and > 65 pg/ml, respectively. Patients with MCI were classified as negative for CSF biomarkers (all of them were normal) or positive for CSF biomarkers (all of them were positive). Patients with some positive CSF biomarkers were not considered in the study to avoid difficulties in interpreting the results.

### Total RNA extraction and cDNA synthesis

Total RNA was extracted from the plasma samples by using the *mir*Vana™ PARIS™ RNA and Native Protein Purification Kit (Cat. No. AM1556, Thermo Fisher Scientific) according to the manufacturer’s instructions. Briefly, 300 μl plasma was added to an equal volume of 2× denaturing solution and then spiked with 10 μl of 100 pM synthetic cel-miR-39-3p (478293_mir, Thermo Fisher Scientific). After phenol extraction, total RNA was eluted in 40 μl of 95 °C nuclease-free water following the recommended protocol. The cDNA template was prepared using the TaqMan™ Advanced miRNA cDNA Synthesis Kit (Cat No. A25576, Applied Biosystems) and according to the corresponding user guide (publication number MAN0016122, revision C.0) using 2 μl of sample eluent. The pre-amplified cDNA product was stored at − 20 °C until ready for final detection by RT-PCR.

### Expression profiling of miRNAs through TaqMan microarray analysis

The expression profile of miRNAs in 19 plasma samples was carried out by loading a 1:10 dilution of pre-amplified cDNA for each sample along with TaqMan® Fast Advanced Master Mix into two microarray cards (TaqMan® Advanced miRNA Human A and B cards, Applied Biosystems), each containing 384 assays. For these arrays, there is a *n* = 1 technical replicate for each RNA probe. The cards were run on a QuantStudio™ 7 Flex RT-PCR System (Life Technologies) and amplified based on the corresponding user guide (publication number MAN0016122, revision C.0). The threshold values were determined by the QuantStudio™ software v-1.3. The data were imported into Thermo Fisher cloud, and their quality was evaluated based on the following criteria: (1) RT-PCR products were considered below the detection threshold and deleted if the cycle threshold (Ct) ≥ 35 or if the Ct value were reported as “undetected” and (2) RT-PCR products with an acceptable Ct range (X-35) but an irregular amplification curve were excluded (Suppl Figs. 1 and 2).

### Identification of ECs

To choose the best EC candidates in the plasma samples, two different approaches were applied. First, miRNAs with the least variability within the screening study population were selected by the mean-centering method. In this approach, the data were normalized to the global mean (averaged Ct value of all analyzed miRNAs) to select the miRNA with the smallest standard deviation. Second, the concordance correlation restricted (CCR) normalization procedure was used. This method is an extension of mean-centering and is based on the identification of a restricted number of normalizer miRNAs that closely follow the mean of the expressed miRNAs [8].

### Stability analysis of ECs

The analysis of expression stability was performed by using GeNorm [16] and BestKeeper [17] algorithms. In the GeNorm program, gene stability is assessed by computing the average pairwise variation of a particular reference gene from all other reference genes (*M* value), with lower *M* values indicating greater stability. The BestKeeper algorithm assesses stability by calculating the SD for each gene of interest and Pearson’s coefficient of correlation for each pair of candidate genes. Values of *P* closer to 1.0 indicate greater stability. Ranking results were obtained from each method, and their concordance was compared (Suppl Fig. 3).

### Study of selected ECs by RT-qPCR and assessment of their stability in an independent cohort

The most stable miRNAs selected from the microarray experiment were validated in a new and independent cohort containing physiologically and cognitively confirmed nondemented controls, patients with MCI with or without AD pathology in CSF, and AD patients (*n* = 126). RT-PCR was carried out by using individual TaqMan™ Advanced miRNA Assays and TaqMan® Fast Advanced Master Mix loaded on 384-well plates (Applied Biosystems). The samples were run in duplicate for each assay (EC). The Ct distribution of miRNAs is shown in Suppl Fig. 4. The stability of the EC candidates was assessed using the GeNorm, BestKeeper, and NormFinder algorithms. The NormFinder program allows us to estimate not only the overall expression variation of the candidate genes but also takes into account both intra- and intergroup variations. Genes with the lowest stability value are the most stable. The overall performance of the ECs was evaluated by combining the results of the three analyses.

### Statistical analyses

Quantitative variables are shown as the mean (standard deviation) or median [interquartile range] according to the normality of the data. Absolute and relative frequencies were used to describe qualitative variables. We compared patient characteristics according to diagnostic groups. The Kruskal-Wallis test was used to compare quantitative variables, and the chi-squared test was used for qualitative variables. All statistical analyses and data processing procedures were performed using the R software, version 3.5.2 (Vienna, Austria).

## Results

### Patient characteristics

The screening study consisted of 19 females with a diagnosis of mild to moderate AD with confirmed pathological levels of Aβ42 in CSF (≤ 600 pg/ml) and MMSE score ≥ 20 (Table 1). The stability assessment cohort consisted of 126 individuals as follows: 24 subjects without AD or any kind of dementia and negative for core CSF biomarkers of AD, 25 subjects with MCI and negative for core CSF biomarkers (MCI −), 22 subjects with MCI and positive for CSF biomarkers (MCI +), and 55 subjects with AD and positive for CSF biomarkers. As displayed in Table 1, there was a significant difference in age between the groups (*P* = 0.001) in the stability assessment study. In addition, as expected, there was a significant difference between the groups in MMSE scores (*P* < 0.001) and CSF biomarker values (*P* < 0.001). The subjects included in the validation cohort did not show significant differences in clinical variables (Table 1).

**Table 1:** The demographic characteristics, clinical comorbidities, and biomarker results of the study population

	Screening study	Stability assessment study				
	AD, *N* = 19, mean (SD), median [IQR] or *n* (%)	CTL, *N* = 24, median [IQR] or *n* (%)	MCI −, *N* = 25, median [IQR] or *n* (%)	MCI +, *N* = 22, median [IQR] or *n* (%)	AD, *N* = 55, median [IQR] or *n* (%)	*P* value
**Sociodemographic characteristics**						
Sex, female	19 (100%)	16 (66.7%)	15 (60.0%)	11 (50.0%)	23 (41.8%)	0.096
Age, years	75.2 (5.68)	68.0 [62.5; 73.0]	69.0 [63.0; 74.0]	73.0 [70.0; 77.0]	73.0 [68.7; 79.0]	0.001
**Alzheimer parameters**						
MMSE		30.0 [28.8; 30.0]	25.0 [23.0; 27.0]	27.0 [25.0; 28.0]	24.0 [22.0; 25.5]	< 0.001
Aβ42	484 [382; 532]	1086 [972; 1419]	846 [689; 960]	491 [422; 541]	430 [340; 530]	< 0.001
T-tau	601 [494; 734]	298 [222; 416]	237 [193; 265]	638 [530; 728]	563 [335; 846]	< 0.001
P-tau	91.0 [76.8; 114]	52.8 [43.8; 62.3]	43.7 [38.5; 48.4]	85.8 [77.5; 111]	80.4 [55.0; 104]	< 0.001
**Comorbidities**						
Depression	7 (36.8%)	7 (29.2%)	11 (44.0%)	7 (31.8%)	16 (29.1%)	0.588
Hypertension	13 (68.4%)	8 (33.3%)	14 (56.0%)	8 (36.4%)	30 (54.5%)	0.184
Diabetes mellitus	3 (15.8%)	5 (20.8%)	6 (24.0%)	2 (9.09%)	10 (18.2%)	0.593
Dyslipidemia	8 (42.1%)	10 (41.7%)	9 (36.0%)	10 (45.5%)	19 (34.5%)	0.807

### Selection of candidate ECs

Among all miRNAs analyzed by TaqMan low-density array cards, hsa-miR-324-5p, hsa-miR-22-5p, hsa-miR-103a-2-5p, hsa-miR-362-5p, hsa-miR-425-3p, hsa-miR-423-5p, hsa-let-7i-3p, hsa-miR-532-5p, and hsa-miR-1301-3p were identified as the miRNAs with the least variability among the study population after applying the mean-centering and CCR methods. These miRNAs were also highly expressed in all samples and therefore were selected as the most suitable EC candidates (Suppl Table 1). The expression stability of the selected candidates was characterized by GeNorm and BestKeeper algorithms, and the candidates were ranked based on their stability scores in both algorithms (Suppl Table 1).

### Stability study of EC candidates by RT-qPCR in an independent cohort

**Fig. 1:**
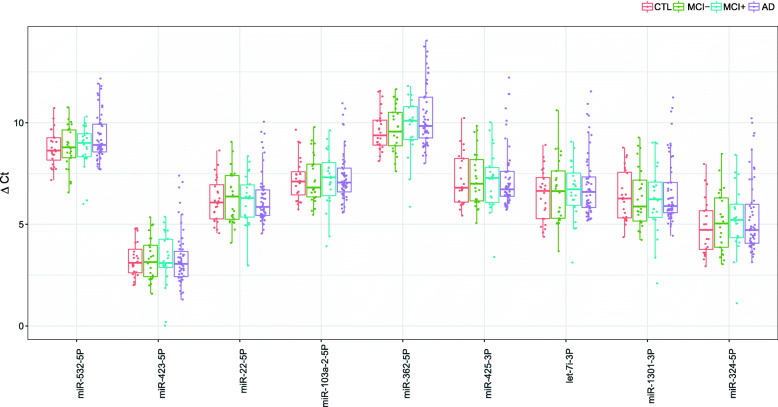
The 9 EC candidates in the validation cohort subjects showed a low Ct

**Fig. 2:**
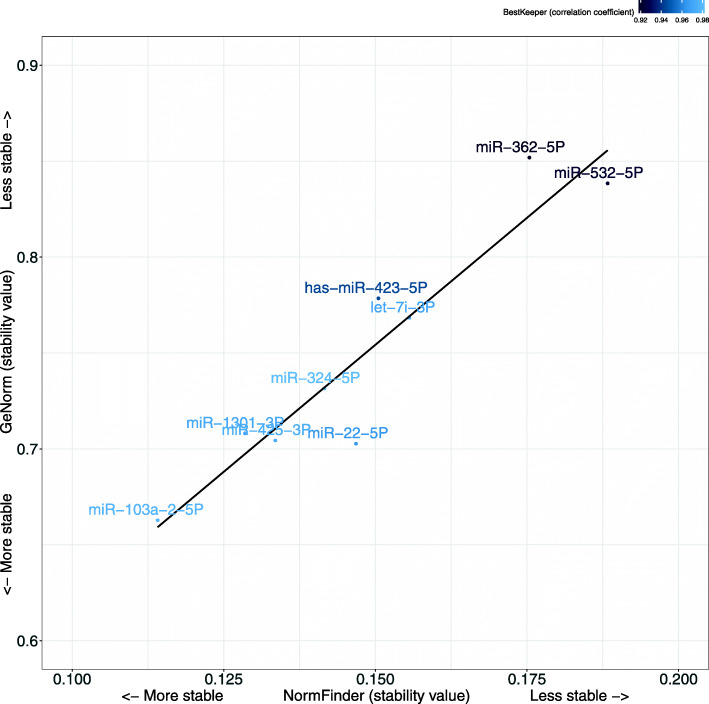
Stability study of the microRNAs studied according to the GeNorm, BestKeeper, and NormFinder algorithms

An independent cohort consisting of controls, MCI patients with or without changes in pathological CSF biomarkers of AD, and probable AD patients was used for the validation of 9 EC candidates selected from the screening study. A low Ct was observed for all 9 EC candidates in the validation cohort subjects (Fig. 1). In addition, we did not observe any significant differences in the expression levels of EC candidates between the four groups (control, MCI −, MCI +, and AD) (Table 2). Among these 9 ECs, hsa-miR-103a-2-5p, hsa-miR-22-5p, hsa-miR-1301-3p, and hsa-miR-425-3p were identified as the most stable by the GeNorm, BestKeeper, and NormFinder algorithms. However, hsa-miR-423-5p, hsa-miR-532-5p, and hsa-miR-362-5p showed less stability (Table 2). Therefore, our results identified hsa-miR-103a-2-5p as the best EC, followed by hsa-miR-22-5p, hsa-miR-1301-3p, and hsa-miR-425-3p, which had similar stability values for all three methods (Fig. 2).

**Table 2:** The most stable miRNAs in the plasma samples of subjects included in the stability assessment study were analyzed by the GeNorm, BestKeeper, and NormFinder algorithms

miRNAs	GeNorm, *M* value	Bestkeeper, *R*	NormFinder, stability value	ΔCt, mean (SD)	Comparison between diagnostic groups, *P* value
miR-103a-2-5P	0.66	0.98	0.11	7.28 (1.22)	0.965
miR-22-5P	0.70	0.97	0.15	6.25 (1.22)	0.997
miR-1301-3P	0.71	0.97	0.13	6.44 (1.53)	0.955
miR-425-3P	0.70	0.97	0.13	7.23 (1.42)	0.995
miR-324-5P	0.73	0.98	0.14	5.19 (1.61)	0.639
let-7i-3P	0.77	0.97	0.16	6.72 (1.49)	0.785
has-miR-423-5P	0.78	0.94	0.15	3.29 (1.20)	0.961
miR-532-5P	0.84	0.92	0.19	9.01 (1.10)	0.294
miR-362-5P	0.85	0.92	0.18	9.97 (1.38)	0.211

## Discussion

In the present study, we identified the nine most stable miRNAs in a cohort of patients with AD. Then, we assessed the stability of the selected miRNAs in an independent cohort consisting of controls, MCI patients with or without pathological evidence of AD in CSF, and probable AD patients. From this profile, we selected four miRNAs (hsa-miR-103a-2-5p, hsa-miR-22-5p, hsa-miR-1301-3p, and hsa-miR-425-3p) that demonstrated better stability for use as endogenous controls in cognitive impairment miRNA studies.

Changes in miRNA expression have been associated with various pathological processes, including pathological events in AD [12]. These small molecules are detectable in the circulatory system and have shown high stability; therefore, their use as safe biomarkers has attracted much attention in recent years. RT-qPCR is one of the most commonly used approaches for the quantification of miRNA expression. However, because of its high sensitivity, the accurate interpretation of the results depends on the use of appropriate and stable ECs for data normalization to minimize nonbiological variations between samples. The most common normalization strategies used in RT-qPCR analysis of circulating miRNAs are the use of exogenous oligonucleotides (such as cel-miR-39 from *Caenorhabditis elegans*), mean-centering, and endogenous miRNAs [7, 18]. Several studies have used exogenous oligonucleotides for normalization of the data [19]. However, exogenous controls only correct RT-qPCR data in terms of the variability related to RNA extraction and reverse transcription because they are added to the biological samples just before RNA extraction. Other variables, such as collection and storage conditions or the number of freeze and thaw cycles of the samples, that affect the final results cannot be corrected by the use of exogenous controls. In this regard, endogenous miRNAs might be considered optimal reference genes since their expression is affected by the same variables as the expression of target genes. Therefore, when a limited number of miRNAs are analyzed, identifying stable endogenous controls is crucial for data normalization. In a study by Nagaraj et al. [20], the RT-qPCR data from the validation study were normalized by using the five most stable miRNAs that were found in the pilot study. They identified hsa-miR-185-5p, hsa-miR-128-3p, hsa-miR-130b-3p, hsa-miR-15a-5p, and hsa-miR-425-3p as the most stable miRNAs in plasma samples of controls and AD patients. Among these five miRNAs, hsa-miR-425-3p was also found to be a stable miRNA in our study. Their pilot study consisted of 20 subjects (7 AD, 7 MCI +, and 6 controls); however, they did not verify the stability of these five miRNAs in a larger cohort. In another study by Siedlecki-Wullich et al. [21], hsa-miR-191-5p and hsa-miR-484 were identified as the most stable reference genes in the plasma samples of 38 healthy controls, 26 MCI, 56 AD, and 27 frontotemporal dementia (FTD) patients. The authors used the geometric mean of hsa-miR-191-5p and hsa-miR-484 for the normalization of their data. In the study by Kumar and Reddy [22], hsa-miR-106a was identified as the least variable miRNA in the plasma samples of 11 AD, 9 MCI, and 20 control subjects, and this miRNA jointly with Ath-159a (exogenous control) was used for normalization of the validation study.

The discrepancies between our results and the results of previous studies and between those studies may be attributed to the use of different technologies for the evaluation of miRNA expression and/or the use of different algorithms for the evaluation of miRNA stability. For example, Nagaraj et al. used Exiqon technology for RNA extraction, cDNA synthesis, and RT-qPCR and the NormFinder algorithm to assess the stability of miRNAs [20]. Kumar et al. used NanoString technology to evaluate the miRNA expression and global rank-invariant set normalization (GRSN) [23] to assess miRNA stability [22]. Siedlecki-Wullich and colleagues used the Qiagen Kit for RNA isolation and TaqMan technology for cDNA synthesis and miRNA expression profiling and the NormFinder algorithm for the assessment of miRNA stability [21]. In the present study, we used TaqMan technology for the evaluation of miRNA expression and three different algorithms, GeNorm, BestKeeper, and NormFinder, for the stability evaluation of the miRNAs. Therefore, this highlights the importance of the use of the unified methodology in miRNA expression assessment studies to achieve comparable results between studies. In addition, none of the aforementioned studies validated their identified stable reference miRNAs in an independent cohort. Therefore, to the best of our knowledge, no previous study has been performed to identify and validate appropriate reference genes in the plasma samples of patients with AD. Here, we present hsa-miR-103a-2-5p, hsa-miR-22-5p, hsa-miR-1301-3p, and hsa-miR-425-3p as suitable ECs for data normalization of plasmatic miRNAs in studies of cognitive impairment.

The main strength of our study is that our validation cohort consisted of a diverse range of patients who normally attend to a memory clinic. In this population, we included subjects without pathological and clinical symptoms of AD or any other dementia, MCI patients with clinical symptoms of cognitive deterioration that were negative for AD core CSF biomarkers, MCI patients who were positive for all three AD biomarkers in CSF and with clinical manifestations of cognitive decline, and patients with probable AD. Therefore, we can conclude that the stability of these four EC miRNAs (hsa-miR-103a-2-5p, hsa-miR-22-5p, hsa-miR-1301-3p, and hsa-miR-425-3p) is not affected by either pathological events or clinical manifestations of the disease. Another strength of this study is that the stability of the EC candidates is not affected by age. Accordingly, we observed only week correlations with the age (Suppl Fig. 5), allowing these miRNAs to remain stable independently of the differences in the mean age between the groups. Furthermore, we evaluated the stability of the EC candidates by three different algorithms, while in other studies, the stability was assessed by only one [20] or two methods [24, 25]. This study has some limitations. First, our screening study included only female individuals. Because of the small number of this population, we decided to eliminate possible variabilities regarding gender. However, in the stability assessment study, we included male subjects and different ranges of subjects regarding pathological events and clinical symptoms of AD to minimize the effect of these variables on the selected ECs. Furthermore, this cohort consisted of only controls, MCI with and without amyloid deposition, and AD patients. So, the results will be useful for those patients with suspected AD pathology in their different stages, but they cannot be applied in the study of other types of cognitive impairment.

## Conclusions

In summary, for the first time, we report a profile of four miRNAs, hsa-miR-103a-2-5p, hsa-miR-22-5p, hsa-miR-1301-3p, and hsa-miR-425-3p, as potential plasma ECs to be used for normalization of miRNA expression data in studies of subjects with cognitive impairment. We propose the use of unified pre-experimental, experimental, and data analysis platforms for the evaluation of miRNAs to achieve comparable results between studies of the same types of sample. However, we emphasize that these ECs may not be useful for the normalization of data in studies with different types of samples, methodologies, and technologies from those used here.

## Supplementary Information

**Additional file 1: Suppl Table 1.** The most stable miRNAs in plasma samples of subjects included in the screening cohort. **Suppl Figure 1.** TaqMan Low Density Array determinations quality control. Number of determinations/missings. **Suppl Figure 2.** Ct distribution of miRNAs in TaqMan Low Density Array. **Suppl Figure 3.** The expression stability of the selected candidates characterized by means of GeNorm and Bestkeeper. **Suppl Figure 4.** Ct distribution of miRNAs in *RT-qPCR cohort*. **Suppl Figure 5.** Spearman’s correlations between the selected miRNAs and age.

## Data Availability

The data reported in this manuscript are available within the article and/or its supplementary data. Additional data will be shared upon request by any qualified investigator.

## Abbreviations

miRNAs: MicroRNAs
mRNA: Messenger RNA
UTR: Untranslated region
RT-qPCR: Real-time polymerase chain reaction
AD: Alzheimer’s disease
Aβ42: Amyloid-β42 protein
PET: Proton emission tomography
CSF: Cerebrospinal fluid
MCI: Mild cognitive impairment
MMSE: Mini-Mental State Examination
MCI −: MCI and negative for CSF biomarkers
MCI +: MCI and positive for CSF biomarkers
NIA-AA: National Institute on Aging and Alzheimer’s Disease Association
CT: Computed tomography
MRI: Brain magnetic resonance imaging
EC: Endogenous control
FTD: Frontotemporal dementia
