# Supplementary material for: Identification and validation of endogenous control miRNAs in plasma samples for normalization of qPCR data for Alzheimer’s disease
Source: Alzheimers Res Ther. 2020 Dec 5;12:163. doi: 10.1186/s13195-020-00735-x (PMC7719248; doi:10.1186/s13195-020-00735-x)

**Identification and validation of endogenous control miRNAs in plasma samples for normalization of RT-qPCR data for Alzheimer’s disease**

Dakterzada, F.^1*^; Targa, A.^2,3*^; Benítez, I.D.^2,3^; Romero L.^1^; de Gonzalo-Calvo, D.^2^; Torres G.^2^; Moncusí-Moix, A.^2,3^; Huerto R.^1^; Sánchez-de-la-Torre, M.^2,4^; Barbé, F.^2,3^; Piñol-Ripoll, G.^1**^

^1^ Unitat Trastorns Cognitius, Clinical Neuroscience Research, Santa Maria University Hospital, IRBLleida, Lleida, Spain.

^2^ Translational Research in Respiratory Medicine, Hospital Universitari Arnau de Vilanova-Santa Maria, IRBLleida, Lleida, Spain.

^3^ Centro de Investigación Biomédica en Red de Enfermedades Respiratorias (CIBERES), Madrid, Spain.

^4^ Group of Precision Medicine in Chronic Diseases, Hospital Universitari Arnau de Vilanova-Santa Maria, IRBLleida, Lleida, Spain.

* Co-first authors. FD and AT contributed equally to this study.

**** Corresponding author:**

Gerard Piñol-Ripoll

Cognitive Disorders Unit

Hospital Universitari Santa Maria.

Rovira Roure nº 44. 25198. Lleida. Spain

Telephone: 34-937-727222. Ext. 173. Fax: 34-976-727366

E-mail: [gerard_437302@hotmail.com](mailto:gerard_437302@hotmail.com)

Suppl Table 1. The most stable miRNAs in plasma samples of subjects included in the screening cohort.

Suppl Figure 1. TaqMan Low Density Array determinations quality control. Number of determinations/missings.


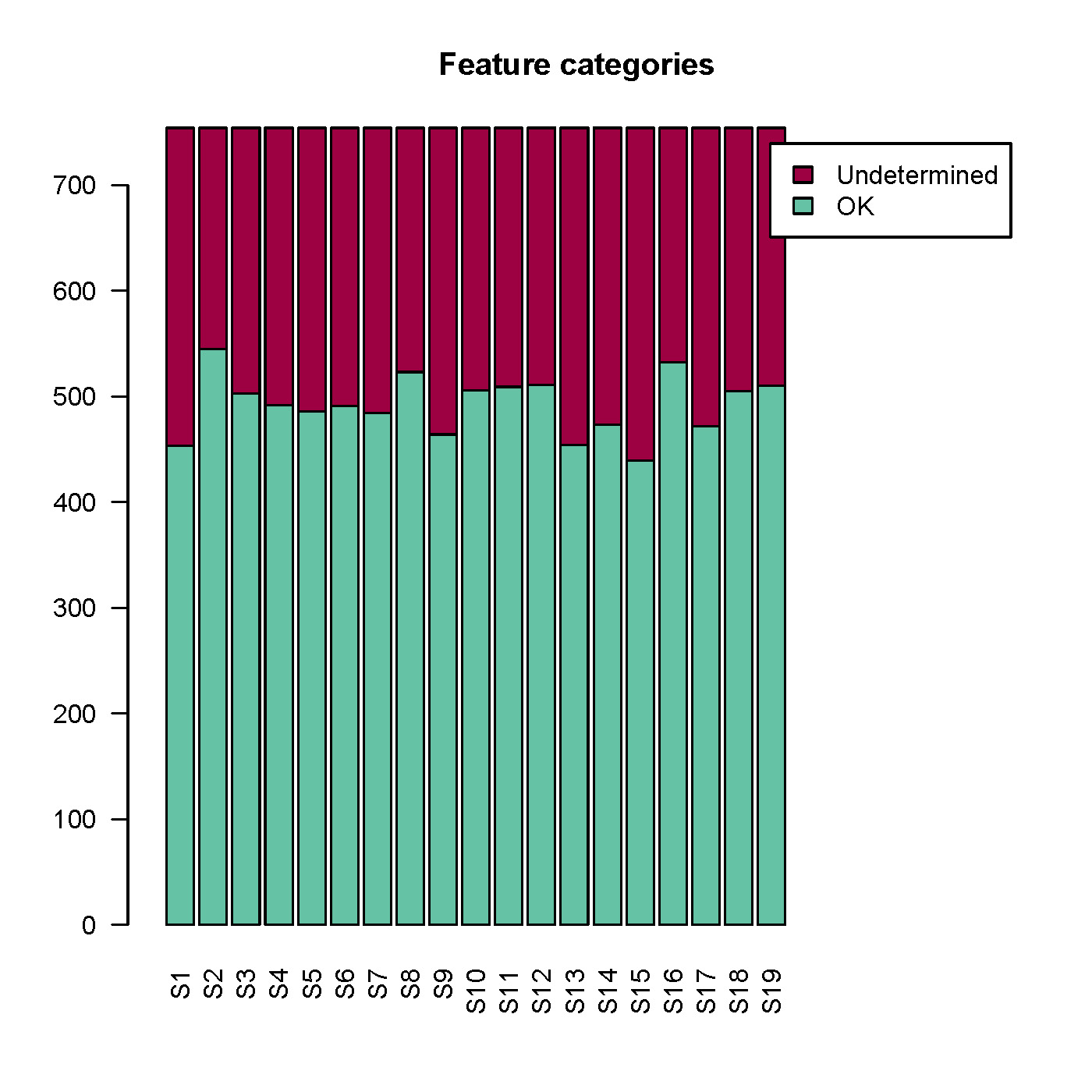


Suppl Figure 2. Ct distribution of miRNAs in TaqMan Low Density Array.


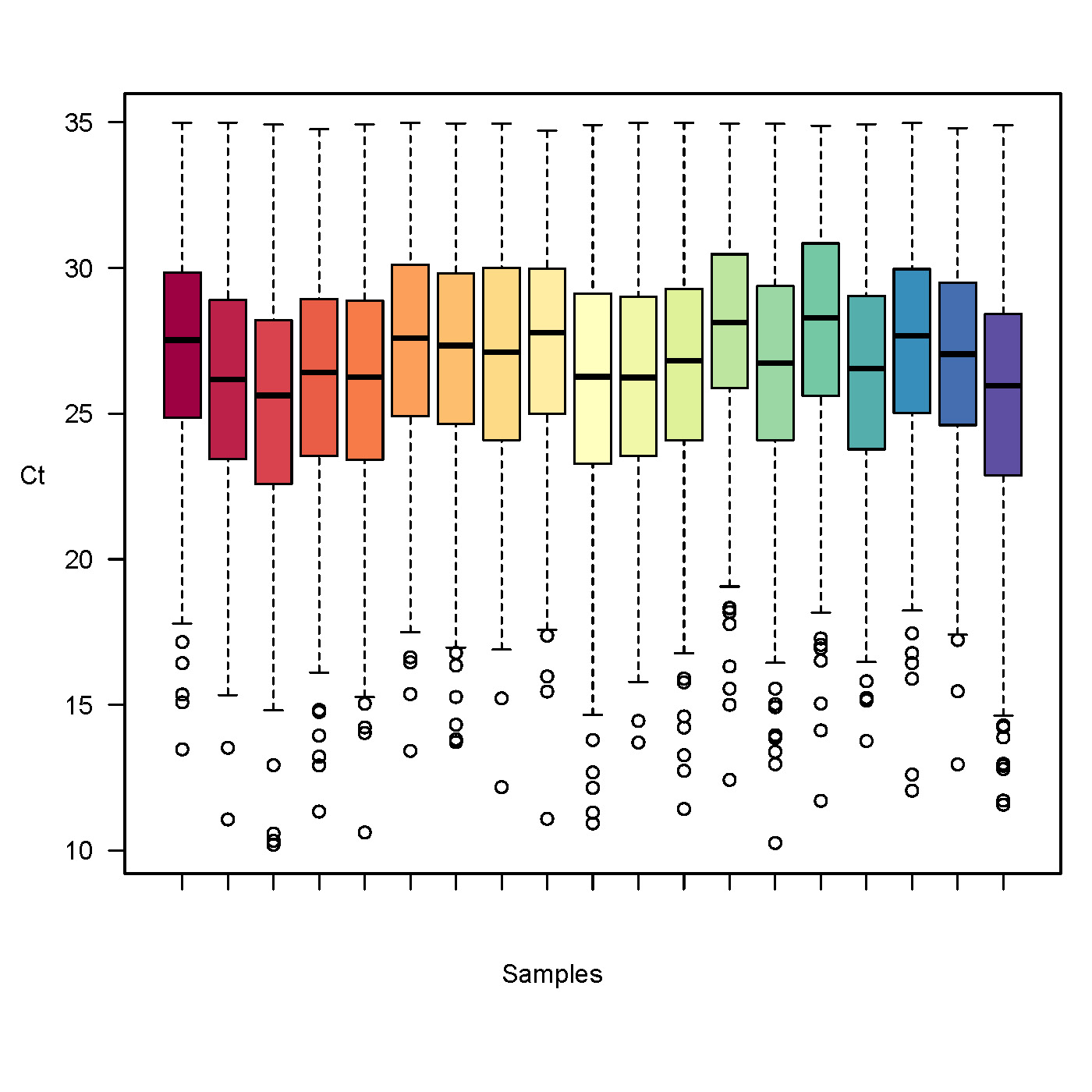


Suppl Figure 3. The expression stability of the selected candidates characterized by means of GeNorm and Bestkeeper.


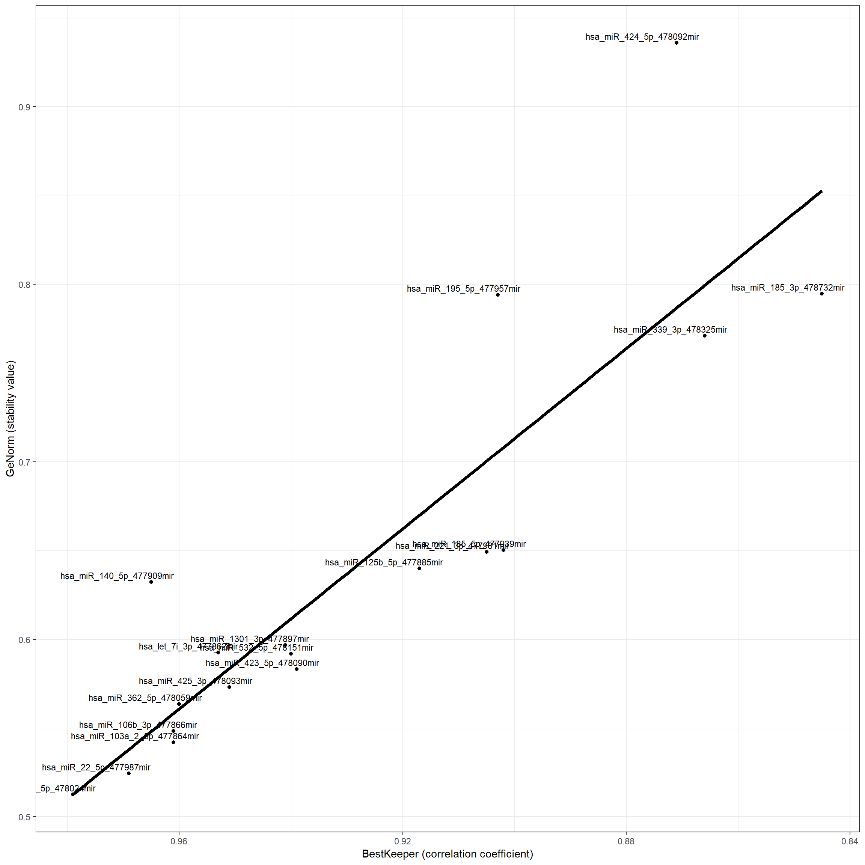


Suppl Figure 4. Ct distribution of miRNAs in *RT-qPCR cohort*.


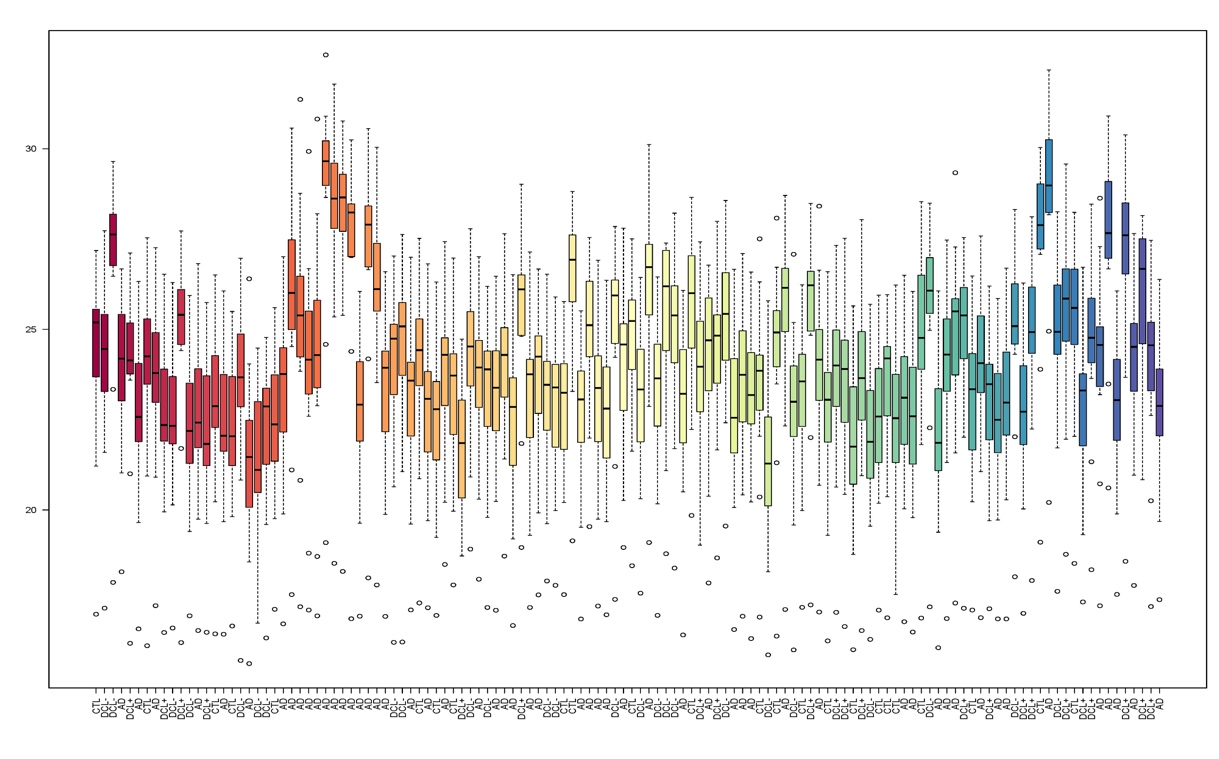


Suppl Figure 5. Spearman’s correlations between the selected miRNAs and age.


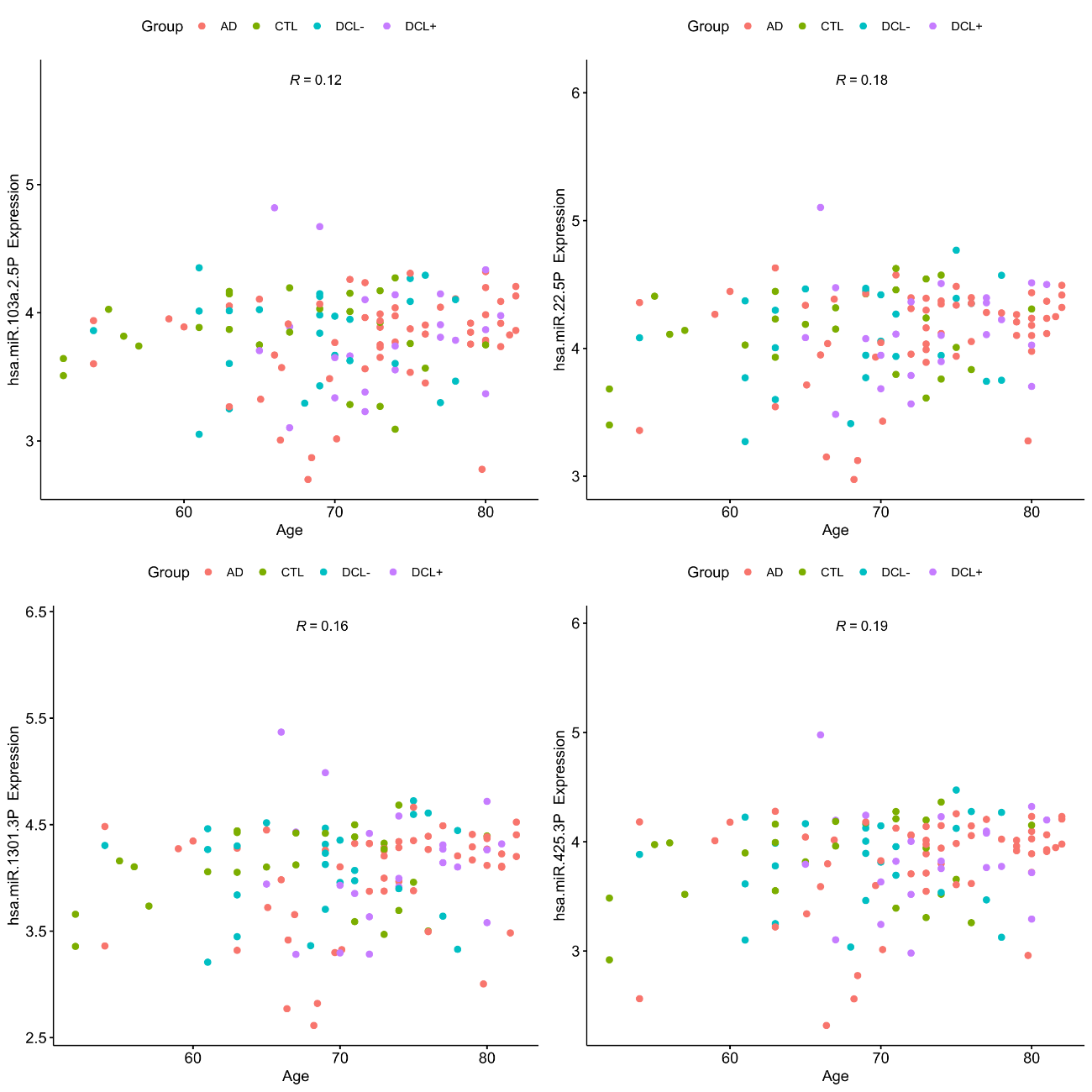

Supplement: Supplementary file 1 — Additional file 1: Suppl Table 1. The most stable miRNAs in plasma samples of subjects included in the screening cohort. Suppl Figure 1. TaqMan Low Density Array determinations quality control. Number of determinations/missings. Suppl Figure 2. Ct distribution of miRNAs in TaqMan Low Density Array. Suppl Figure 3. The expression stability of the selected candidates characterized by means of GeNorm and Bestkeeper. Suppl Figure 4. Ct distribution of miRNAs in RT-qPCR cohort. Suppl Figure 5. Spearman’s correlations between the selected miRNAs and age. [file 13195_2020_735_MOESM1_ESM.docx]
